# Postnatal Sox6 Regulates Synaptic Function of Cortical Parvalbumin-Expressing Neurons

**DOI:** 10.1523/JNEUROSCI.0021-21.2021

**Published:** 2021-10-27

**Authors:** Hermany Munguba, Bidisha Chattopadhyaya, Stephan Nilsson, Josianne N. Carriço, Fatima Memic, Polina Oberst, Renata Batista-Brito, Ana Belen Muñoz-Manchado, Michael Wegner, Gordon Fishell, Graziella Di Cristo, Jens Hjerling-Leffler

**Affiliations:** ^1^Laboratory of Molecular Neurobiology, Department Medical Biochemistry and Biophysics, Karolinska Institutet, Stockholm SE-17177, Sweden; ^2^Department of Neurosciences, Université de Montréal and Centre de Recherche, Centre Hospitalier Universitaire Ste-Justine, Montréal, Québec, Canada; ^3^Rose F. Kennedy Center, Albert Einstein College of Medicine, New York, NY 10461; ^4^Universidad de Cádiz, 11003 Cadiz, Spain; ^5^Institut für Biochemie, Emil-Fischer-Zentrum, Friedrich-Alexander-Universität Erlangen-Nürnberg, 91054 Erlangen, Germany; ^6^Harvard Medical School Boston, Massachusetts, MA 02115; ^7^Stanley Center at the Broad, Cambridge, Massachusetts, MA 02142

**Keywords:** axonal boutons, postnatal maturation, Pvalb-expressing neurons, Sox6, synaptic function, TrkB

## Abstract

Cortical parvalbumin-expressing (Pvalb^+^) neurons provide robust inhibition to neighboring pyramidal neurons, crucial for the proper functioning of cortical networks. This class of inhibitory neurons undergoes extensive synaptic formation and maturation during the first weeks after birth and continue to dynamically maintain their synaptic output throughout adulthood. While several transcription factors, such as Nkx2-1, Lhx6, and Sox6, are known to be necessary for the differentiation of progenitors into Pvalb^+^ neurons, which transcriptional programs underlie the postnatal maturation and maintenance of Pvalb^+^ neurons' innervation and synaptic function remains largely unknown. Because Sox6 is continuously expressed in Pvalb^+^ neurons until adulthood, we used conditional knock-out strategies to investigate its putative role in the postnatal maturation and synaptic function of cortical Pvalb^+^ neurons in mice of both sexes. We found that early postnatal loss of Sox6 in Pvalb^+^ neurons leads to failure of synaptic bouton growth, whereas later removal in mature Pvalb^+^ neurons in the adult causes shrinkage of already established synaptic boutons. Paired recordings between Pvalb^+^ neurons and pyramidal neurons revealed reduced release probability and increased failure rate of Pvalb^+^ neurons' synaptic output. Furthermore, Pvalb^+^ neurons lacking Sox6 display reduced expression of full-length tropomyosin-receptor kinase B (TrkB), a key modulator of GABAergic transmission. Once re-expressed in neurons lacking Sox6, TrkB was sufficient to rescue the morphologic synaptic phenotype. Finally, we showed that Sox6 mRNA levels were increased by motor training. Our data thus suggest a constitutive role for Sox6 in the maintenance of synaptic output from Pvalb^+^ neurons into adulthood.

**SIGNIFICANCE STATEMENT** Cortical parvalbumin-expressing (Pvalb^+^) inhibitory neurons provide robust inhibition to neighboring pyramidal neurons, crucial for the proper functioning of cortical networks. These inhibitory neurons undergo extensive synaptic formation and maturation during the first weeks after birth and continue to dynamically maintain their synaptic output throughout adulthood. However, it remains largely unknown which transcriptional programs underlie the postnatal maturation and maintenance of Pvalb^+^ neurons. Here, we show that the transcription factor Sox6 cell-autonomously regulates the synaptic maintenance and output of Pvalb^+^ neurons until adulthood, leaving unaffected other maturational features of this neuronal population.

## Introduction

Parvalbumin-expressing (Pvalb^+^) neurons comprise the largest class of GABAergic interneurons in the cortex. Their birth obeys a dorsal-ventral temporal distribution within the medial ganglionic eminence (MGE; Wonders et al., 2008; Inan et al., 2012), followed by a coordinated migration pattern and subsequent allocation to the different cortical layers (Anderson et al., 1997, 2001; Bartolini et al., 2013). In mice, during the second postnatal week Pvalb^+^ neurons undergo prominent transcriptional changes that mark the beginning of their electrophysiological, molecular and synaptic maturation (Micheva and Beaulieu, 1996; Chattopadhyaya et al., 2004; Okaty et al., 2009; Goldberg et al., 2011). After approximately three postnatal weeks, neurons ultimately acquire their hallmark characteristics, such as high-frequency action potentials (APs), Pvalb expression itself, robust somatic innervation of neighboring pyramidal neurons, as well as a specialized extracellular matrix, known as perineuronal nets (PNNs; Hu et al., 2014).

Neuronal maturation involves reaching several developmental milestones while retaining plasticity to adapt to new stimuli (Takesian and Hensch, 2013). For example, alterations in local network activity can trigger transient changes in Pvalb^+^ neurons' firing properties (Dehorter et al., 2015), PNNs (Nowicka et al., 2009; Banerjee et al., 2017; Favuzzi et al., 2017), and the strength of synaptic inhibition they provide, thereby rebalancing local levels of excitation and inhibition (Moore et al., 2018). Activity-dependent expression of brain-derived neurotrophic factor (BDNF) is one of the strongest modulators of Pvalb^+^ neurons' maturation and dynamic tuning, acting primarily via the activation of tropomyosin-receptor kinase B (TrkB) in these cells (Hong et al., 2008; Lin et al., 2008; Bloodgood et al., 2013). Nonetheless, the transcriptional programs underlying the postnatal maturation and maintenance of Pvalb^+^ neurons, with few exceptions (Dehorter et al., 2015), are largely unknown.

Loss of the transcriptional factor Sox6 embryonically in GABAergic neurons, via *Lhx6*^Cre^-dependent removal, perturbs layer allocation of MGE-derived neurons and the ectopically located cells show delayed electrophysiological maturation, as well as loss of mature markers, such as Pvalb (Batista-Brito et al., 2009). Ultimately, this early loss of Sox6 leads to lethal epilepsy at postnatal day (P)17–P19 (Batista-Brito et al., 2009), in a period of significant increase of cortical Pvalb^+^ neuron axonal arborization around pyramidal neurons during normal development (Chattopadhyaya et al., 2004).

Because Pvalb^+^ neurons continually express Sox6 after birth, we sought to investigate whether Sox6 plays a specific role in regulating Pvalb^+^ neuron functional maturation postnatally. Here, we show that postnatal loss of Sox6 in Pvalb^+^ neurons, while not affecting several other aspects of their maturation, specifically disrupts synaptic bouton maturation and maintenance by controlling the levels of TrkB expression in this neuronal population.

## Materials and Methods

### Mouse lines

All mouse handlings in this study were according to local ethical regulations and were approved by the local committees for ethical experiments on laboratory animals [Stockholms Norra Djurförsöksetiska nämnd, Sweden, and Comité Institutionnel des Bonnes Pratiques Animales en Recherche (CIBPAR) of Centre Hospitalier Universitaire Ste-Justine Research Center]. We used different Cre-expressing mouse lines to label Pvalb^+^ cells and conditionally remove Sox6 at various time points. We used the *Pvalb*^Cre^ knock-in mice for wide-ranging targeting of Pvalb^+^ neurons after the second postnatal week (Hippenmeyer et al., 2005), and the GAD76-GFP G42 line (which labels a subset of Pvalb^+^ neurons; Chattopadhyaya et al., 2004) was used to target these cells in the adult cortex. Animals were crossed with the reporter mouse line R26R CAG-boosted eGFP (RCE; Miyoshi et al., 2010), together with *Sox6* loxp background (Dumitriu et al., 2006; *Sox6*^fl/+^ or *Sox6*^fl/fl^). Experimental animals included mice of both sexes. We also used CD1 wild-type mice to describe the endogenous expression of Sox6 throughout postnatal maturation and after accelerating rotarod training (P28 and P90).

### Immunohistochemistry

Animals were deeply anesthetized with ketamine/xylazine (4:1) and transcardically perfused with PBS solution followed by ice-cold 4% paraformaldehyde (PFA)/PBS solution. The brains were dissected and postfixed for 1 h in ice-cold 4% PFA/PBS solution. These were then rinsed in PBS and cryo-protected in 4°C 30% sucrose/PBS solution overnight until sinking. Brains were embedded in optimal cutting temperature (OCT, Histolab Products AB) and frozen to −80°C until cryo-sectioned in a Leica cryostat at 10- to 14-µm thickness.

Sections were washed in PBS-Tween (0.1% Tween20 in PBS) and incubated in a blocking solution (2.5% normal goat serum, 2.5% donkey serum, 2.5% BSA, 0.5 m NaCl, and 0.3% Tween 20 in PBS) for 1 h at room temperature. They were then incubated in primary antibodies in dilution buffer (2.5% BSA, 0.5 m NaCl, and 0.3% Tween 20 in PBS) overnight at 4°C, washed in PBS four times for 15 min each and 1 h of secondary antibody incubation at room temperature, followed by four washes in PBS for 10 min each. Nuclear counterstaining was performed with 100 ng/ml of 4,6-diamidino-2-phenylindole (DAPI) solution in PBS for 5 min. Primary antibodies were used at the following concentrations: chicken anti-green fluorescent protein (1:2000, Abcam), mouse anti-Pvalb (1:1000; Sigma-Aldrich), guinea pig anti-Sox6 (1:2000; Stolt et al., 2006), mouse anti-NeuN (1:200; Abcam), and biotinylated Wisteria Floribunda Agglutinin (WFA) to label PNNs (VectorLabs). Secondary antibodies conjugated with Alexa Fluor dyes 488 (1:1000), 555, and 647 (1:400; Invitrogen) or Streptavidin 555 (1:500, Invitrogen) were used to visualize the signals. Sections were then mounted with Fluoromount-G (Southernbiotech).

We used a Carl Zeiss LSM700 or LSM880 confocal microscope with Plan-Apochromat 10× or 100× objectives to acquire images of the primary somatosensory cortex (S1), always spanning all six cortical layers. Cells were then counted using the Cell Counter plugin on ImageJ/FIJI software. For *in vivo* bouton analysis, we measured the diameter of axonal boutons surrounding cell bodies using NeuN^+^ or DAPI to define the cellular circumference.

### *In situ* hybridization and analysis

Mice were deeply anesthetized with ketamine/xylazine (4:1) and brains immediately removed and embedded in an OCT cryomount (Histolab Products AB), frozen on dry ice, sectioned at 10 μm using a cryostat (Leica Biosystems; kept at −80°C). *In situ* hybridization was performed following the manufacturer's instructions for fresh-frozen tissue (RNAscope technology, Advanced Cell Diagnostics) for the following genes: *Pvalb*, *Sox6*, and a custom-made *Ntrk2* (for full-length TrkB), while DAPI was included to determine the different cortical layers.

Confocal images were acquired using Plan-Apochromat 20× or 40× objectives and images were analyzed with Imaris 8.2 (Oxford Instruments). *Pvalb*-channel was used as a mask to define individual cells (cell body selection) and the number of *TrkB-FL* or *Sox6* puncta in Pvalb^+^ neurons was analyzed using the vesicle detection feature. For each mouse, between 50 and 150 *Pvalb*^+^ cell bodies were included in the analysis.

### Acute slice electrophysiology

Whole-cell patch-clamp electrophysiological recordings were obtained from eGFP-expressing cells in acute brain slices prepared from P21 to P32 *Pvalb*^Cre^:RCE animals. Animals were anesthetized deeply with ketamine/xylazine (4:1), decapitated, and the brain was quickly removed and transferred to ice-cold cutting-solution of the following composition: 87 mm NaCl, 75 mm sucrose, 2.5 mm KCl, 25 mm NaHCO_3_, 1.25 mm NaH_2_PO_4_, 7 mm MgCl_2_, 1 mm CaCl_2_, and 10 mm glucose. Animals older than P21 were transcardically perfused with cutting-solution. The brain was then fixed to a stage and 300-μm slices were cut on a vibratome (VT1200 S, Leica). Slices were then individually transferred into an incubation chamber containing oxygenated artificial CSF (aCSF) of the following composition: 125 mm NaCl, 2.5 mm KCl, 25 mm NaHCO_3_, 1.25 mm NaH_2_PO_4_, 2 mm MgCl_2_, 2 mm CaCl_2_, and 10 mm glucose at 35°C for 30 min followed by at least 30 min at room temperature before recordings. During recording, slices were continually perfused with aCSF. Patch electrodes were made from borosilicate glass (resistance 4–8 MΩ; Hilgenberg, GmbH) and filled with a solution containing the following: 135 mm KCl, 10 mm Na-phosphocreatine, 10 mm HEPES, 4 mm Mg-ATP, 0.3 mm Na-GTP, and 5 mg/ml of neurobiotin (VectorLabs).

Paired recordings were performed in LII/III in S1 of *Pvalb*^Cre/+^:RCE^eGFP/+^ control and Sox6-cKO animals. eGFP and pyramidal cells were selected between 10–50 µm of distance from each other. The presynaptic cell in current-clamp received a stimulus eliciting eight APs at 10 Hz, while the postsynaptic cell was held at −70 mV in voltage-clamp.

### Analysis of intrinsic and synaptic properties

Depolarizing and hyperpolarizing current steps were used to extract the following electrical properties of recorded neurons: resting membrane potential (RMP) was measured after membrane rupture; input resistance (iR) was obtained by the steady-state voltage response to a hyperpolarizing current step injection; membrane time constant (τm) was extracted by performing an exponential fit to the decay phase of a voltage response to a negative current step; H-current-mediated sag was measured as the voltage difference between the peak hyperpolarization and the steady-state response to a long (1-s) current step. AP threshold was obtained from the first AP discharge after the minimum current injection to elicit an AP. The additional following parameters were measured from the same protocol: AP amplitude; AP width at half amplitude; and after-hyperpolarization (AHP) latency (the time from spike threshold to lowest point of the AHP) and amplitude (in mV).

### Analysis of perisomatic innervation in organotypic slice

Slice culture preparation was performed using Sox6^fl/fl^ mice pups of either sex, the detailed methods for which have been described elsewhere (Chattopadhyaya et al., 2004). Cortical slices were then placed on transparent Millicell membrane inserts (Millipore), in six-well plates containing culture medium and incubated at 34°C with 5% CO_2_-enriched air for the different developmental time frames. For biolistic transfection with the gene gun (Bio-Rad), gold particles were coated with the specific plasmids as specified in the text, originally generated by subcloning of a 10 kb region of *Gad1* gene promoter by gap repair in front of the GFP coding region in pEGFP (Clontech) and described in full detail in Chattopadhyaya et al. (2004). This 10-kb Gad1 promoter region confers basket cell specificity in transfected cortical slices allowing us to label them (using P_G67_-GFP) and manipulate their function at the single-cell level (using P_G67_-GFP/Cre). The TrkB plasmid (gift from Eero Castren) was subcloned under the same 10 kb-G_67_ vector to generate G_67_-GFP+TrkB and G_67_-GFP+Cre+TrkB for the rescue experiments.

Slices were fixed, freeze-thawed and immunostained with NeuN (mouse monoclonal catalog #MAB377, 1:400, Millipore), as described in Chattopadhyaya et al. (2004). For each experimental group, equal number of basket cells localized in LII/III and L were analyzed. Confocal image stacks of basket cell axon arbors were acquired using a 63× glycerol objective (NA 1.4, Leica) with Leica TCS SP8 at 1-µm steps of at least 50 µm along the *z*-axis. At least three stacks were acquired for each basket cell. Image stacks were traced using the Neurolucida confocal module and analyzed using Neuroexplorer (Microbrightfield). Analysis of basket cell perisomatic innervation and bouton size was performed as described in detail in (Chattopadhyaya et al., 2004, 2013; Baho et al., 2019) where only innervated cells were included in this analysis. Briefly, the complexity of the basket cell Pvab1 axon branches around a pyramidal cell soma was reported as the average number of intersections and bouton density. The number of intersections represented the intersections between a basket cell axon and the Sholl spheres (9 µm, increment of 1 µm) from the center of the pyramidal cell soma. Bouton density around each basket cell represented the total number of GFP+ boutons in a radius of 9 µm from the center of the pyramidal cell soma; 12–24 pyramidal cells were analyzed for each basket neuron. Bouton size was measured by the diameter of a bouton perpendicular to the basket axon around pyramidal cell soma using Leica confocal software. For each confocal stack, we chose at least four complete neuronal somata (identified by NeuN immunolabeling) and measured bouton size of all GFP+ perisomatic boutons. We then calculated the mean of all bouton analyzed for each basket cell.

### Virus injections and cell counting

G42:*Sox6*^fl/+^ or G42:*Sox6*^fl/fl^ mice (three to four months old) were firstly anesthetized in an enclosed chamber in the presence of isoflurane. Subsequently, animals were fixed on a stereotaxic frame connected to a breathing system providing constant flow of oxygen and isoflurane, which kept the animals deeply anesthesized. Animals were constantly and thoroughly monitored for any signs of pain. Lidocaine was locally applied, before hole was drilled. We injected 1.25–1.5 µl of a cocktail of AAV Cre-Recombinase Dependent on GFP^34^ (CRE-DOG^OPT^), together with AAV2/8 Flex-myr-GFP virus (Neurophotonics Platform) in the somatosensory cortex (S1). With this strategy we were able to express Cre only in eGFP-expressing cells and remove *Sox6* in *Sox6*^fl/fl^ animals. Viral expression was allowed for three weeks for subsequent axonal bouton analysis. Brain collection and following analysis were according to the description for immunohistochemistry (for eGFP, NeuN, and Sox6). Confocal images were collected using 63× oil-objective. Images were analyzed using Imaris, with which we were able to measure the diameter of axonal boutons surrounding NeuN^+^ cell bodies.

### Rotarod training

Adult male mice were positioned on a rotarod apparatus, which rotated in accelerating speed from 4 Hz up to 60 Hz within the duration of each session, which had a maximum duration of 360 s). Latency to fall was annotated for each session and animals were put back in their cage for 2–3 min before the start of the next session. Each experimental animal underwent a total of 10 sessions while control animals were littermates and shared home cage with trained animals. Brains were collected 4 h after the last session and processed for *in situ* hybridization as aforementioned.

### Statistics

Statistical tests used and *p* values for each experiment are included in figure legends. Differences were considered statistically significant for *p* < 0.05. In summary, differences between two experimental groups were assessed using two-tailed unpaired Student's *t* test for normally distributed data and Mann–Whitney test for not normally distributed data or Kolmogorov–Smirnov test to compare cumulative distributions. Differences between three or more experimental groups were assessed with one-way ANOVA with Tukey's *post hoc* comparison. Two-way ANOVA (repeated measures) was used when two factors (condition and time) were included in the experiment. All bar graphs represent mean ± SEM. All the statistical analyses were performed using Prism 9.0 (GraphPad Software).

## Results

### Postnatal loss of Sox6 does not affect expression of *Pvalb* and formation of PNNs on Pvalb^+^ neurons

**Figure 1.**
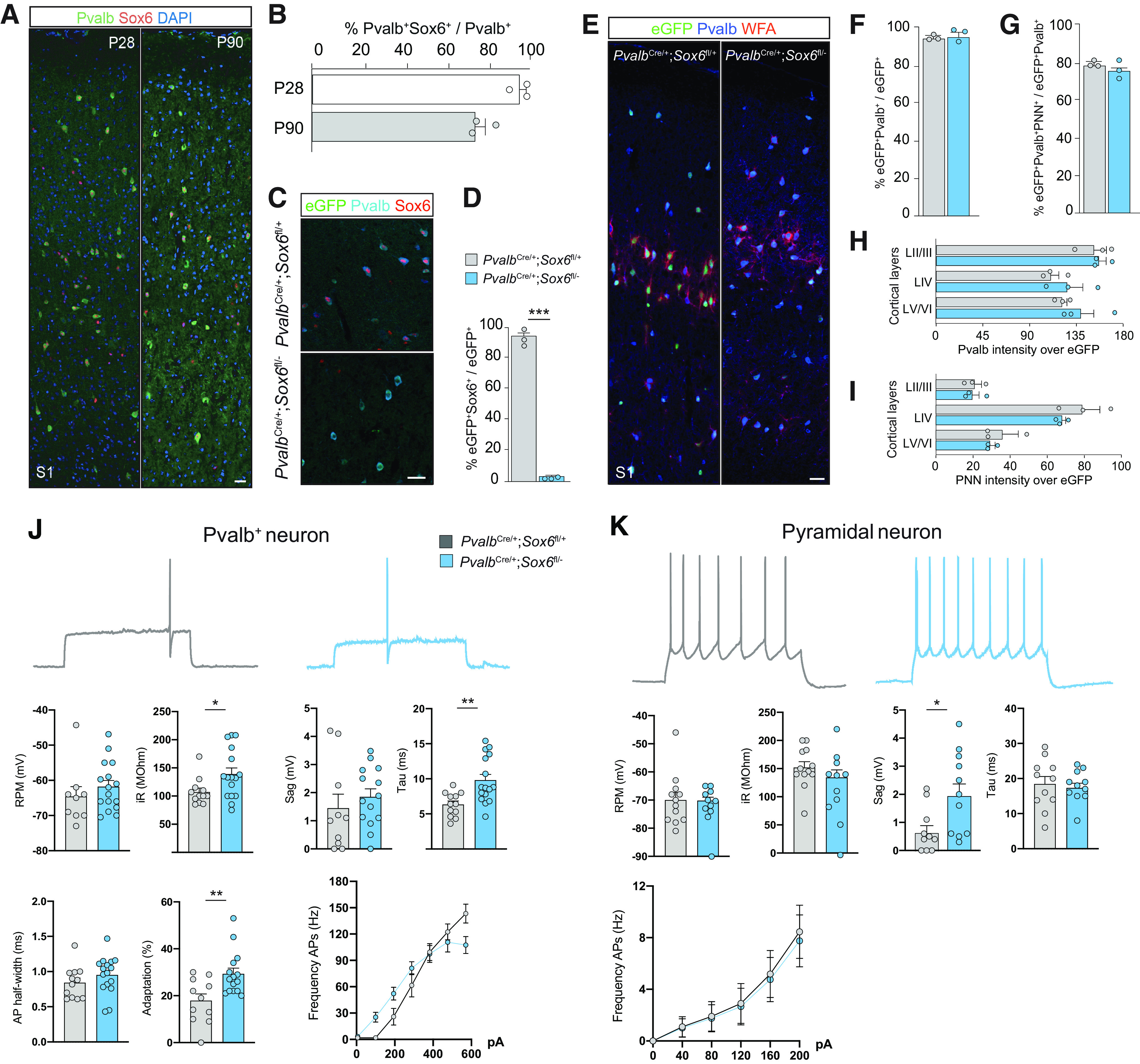
Cortical Pvalb^+^ neurons do not require postnatal expression of Sox6 to achieve their hallmark maturational signature. ***A***, Immunofluorescence of S1 cortical sections (P28 and P90 wild-type mice) showing co-expression of Pvalb and Sox6. ***B***, Bar plots show percentage of co-expressing Pvalb^+^ Sox6^+^ cells. ***C***, Immunofluorescence of S1 cortical sections (P28). ***D***, Bar plot shows efficient Sox6 removal using *Pvalb*^Cre^:RCE: *Sox6*^fl/±^ (*n* = 3 mice per condition; unpaired *t* test, *p* < 0.0001). ***E***, Immunofluorescence of S1 cortical sections (P28) showing eGFP, Pvalb and WFA, which binds to PNNs. ***F***, Bar plots showing percentage of eGFP^+^ cells expressing Pvalb. ***G***, Percentage of eGFP^+^Pvalb^+^ cells enwrapped by PNN/WFA (*n* = 3 mice per condition; unpaired *t* test, *p* = 0.89). ***H***, ***I***, Bar plots display intensity of Pvalb and (***I***) PNN overlapping the eGFP channel throughout cortical layers (*n* = 3 mice per condition; unpaired *t* test, *p* = 0.37). ***J***, Representative current-clamp traces of eGFP^+^ neurons recorded in *Pvalb*^Cre^:RCE: *Sox6*^fl/±^ mice (P21–P32 mice). Frequency of APs after current steps increments of 100 pA [control *n* = 9 cells from 7 mice; Sox6-cKO *n* = 16 from 9 mice; unpaired *t* test, RMP *p* = 0.37; iR *p* = 0.02; Sag *p* = 0.54; Tau *p* = 0.002; AP half-width *p* = 0.16; adaptation *p* = 0.002; frequency APs, two-way ANOVA, repeated measures, row factor (current) *p* < 0.0001; and column factor (genotype), *p* = 0.51]. ***K***, Representative current-clamp traces of pyramidal neurons recorded in *Pvalb*^Cre^:RCE: *Sox6*^fl/±^ mice (P21–P32 mice). Frequency of APs after current steps increments of 40 pA [control *n* = 12 cells from 9 mice; Sox-cKO *n* = 11 from 10 mice; unpaired *t* test, RMP *p* = 0.17; iR *p* = 0.54; Sag *p* = 0.019; Tau *p* = 0.86; frequency APs, two-way ANOVA, repeated measures, row factor (current) *p* < 0.0001; and column factor (genotype), *p* = 0.73]. iR, input resistance; RMP, resting membrane potential: *p < 0.05, **p < 0.01, ***p < 0.001. Error bars, SEM. Scale bar: 20 µm.

Sox6 expression in Pvalb^+^ neurons is maintained from embryonic stages (Batista-Brito et al., 2009) through postnatal development into adulthood (Pvalb^+^Sox6^+^/Pvalb^+^ at P28 94.9 ± 3.2%; at P90 73.9 ± 3.67%; Fig. 1*A*,*B*). To investigate what role Sox6 plays specifically during postnatal maturation of Pvalb^+^ neurons, without affecting its embryonic expression, we used *Pvalb*^Cre^:RCE:Sox6^fl/±^ mice to remove *Sox6* starting at P7-P10 while labeling them with eGFP (Hippenmeyer et al., 2005; Zheng et al., 2011). By P28 the majority of Pvalb^+^ neurons of the S1 express eGFP (85–95%; data not shown), with efficient Sox6 ablation in Pvalb^+^ cells in *Pvalb*^Cre^:RCE:Sox6^fl/–^ (*Sox6*-cKO) mice (unpaired *t* test, *p* < 0.0001; Fig. 1*C*,*D*). We observed no differences in Pvalb^+^ cell density (unpaired *t* test, *p* = 0.89; Fig. 1*E*,*F*) nor Pvalb^+^PNN^+^ co-labeling among eGFP^+^ neurons (unpaired *t* test for *p* = 0.37; Fig. 1*G*), as well as no differences in the levels of Pvalb^+^ and PNN staining intensity across cortical layers at P28 (Fig. 1*H*,*I*). Whole-cell patch-clamp recordings of eGFP^+^ cells in S1 slices of P21–P32 *Pvalb*^Cre^ animals revealed that Sox6-cKO neurons still displayed typical fast-spiking and high-frequency firing properties (Fig. 1*J*), although with slightly altered electrophysiological features (unpaired *t* tests, higher input resistance: *p* = 0.022), faster membrane constant (*p* = 0.002), higher frequency adaptation (*p* = 0.003). Conversely, pyramidal neurons' firing properties were not altered, except for higher Ih-mediated sag (unpaired *t* test, *p* = 0.019; Fig. 1*K*), a possible compensatory mechanism to limit excitability (Fan et al., 2005; van Welie et al., 2004).

### Cortical Pvalb^+^ neurons require Sox6 for axonal maturation and synaptic function

Formation of cortical GABAergic synapses accelerates at the end of postnatal week one and, in particular, axonal trees expand during the entire first postnatal month (Micheva and Beaulieu, 1996; Chattopadhyaya et al., 2004; Pangratz-Fuehrer and Hestrin, 2011). To investigate whether Sox6 activity during postnatal development plays a role in the process of synapse formation and maturation, we used a gene gun transfection approach in cortical organotypic cultures, using a previously characterized plasmid which specifically targets Pvalb^+^ cells (P_G67_; Chattopadhyaya et al., 2004, 2007, 2013; Baho et al., 2019; Di Cristo et al., 2007). This technique allows (1) visualization of Pvalb^+^ basket cells' axonal branching and synaptic innervation onto excitatory neurons during development at high resolution and (2) manipulation of gene expression in isolated Pvalb^+^ neurons in an otherwise wild-type background, allowing for single-cell perturbation and cell-autonomous studies (Baho et al., 2019; Amegandjin et al., 2021).

**Figure 2.**
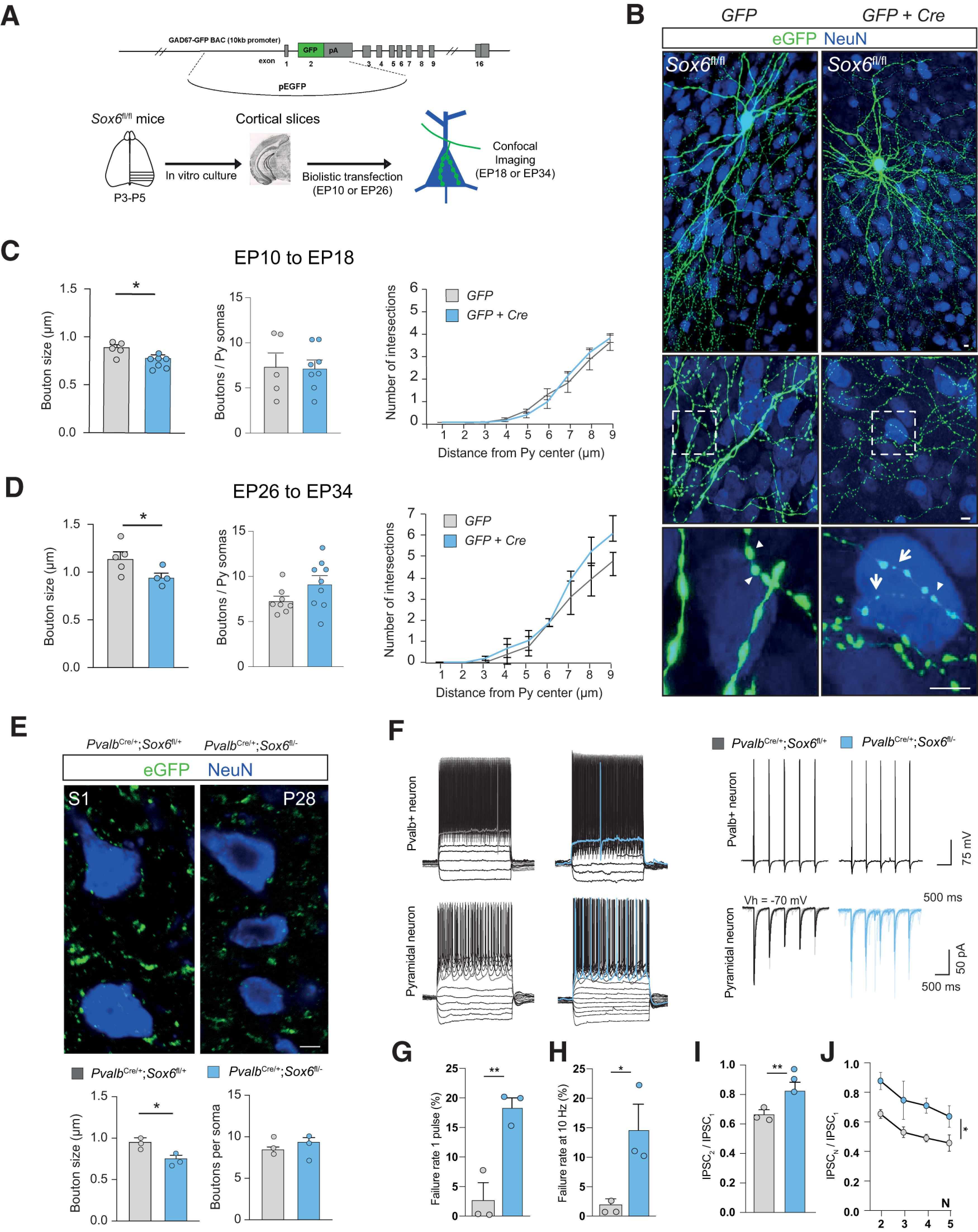
Postnatal loss of Sox6 in Pvalb^+^ basket-cells reduces axonal bouton size and impairs synaptic output. ***A***, Scheme of organotypic slice preparation for analysis of basket cell perisomatic innervation. ***B***, Representative immunofluorescence of EP26–EP34 organotypic slices from Sox6^fl/fl^ mice. Single basket cells were transfected with either only GAD67-*GFP* plasmid or the same plasmid together with *Cre*. ***C***, Mean bouton size (unpaired *t* test with Welch's correction, *p* = 0.0234), bouton density (unpaired *t* test with Welch's correction, *p* = 0.9196) and terminal branching (unpaired *t* test with Welch's correction at all data points *p* > 0.1) around pyramidal somata for individual Pvalb^+^ cells from EP10–EP18 (*n* = 5 P_G67_-*GFP* basket cells vs *n* = 7 P_G67_-*GFP*+*Cre* basket cells). ***D***, Mean bouton size (unpaired *t* test with Welch's correction, *p* = 0.0487), bouton density (unpaired *t* test with Welch's correction, *p* = 0.0934), and terminal branching (unpaired *t* test with Welch's correction at all data points *p* > 0.1) for individual Pvalb^+^ cells around pyramidal somata from EP26 to EP34 (*n* = 8 GFP Ctrl vs *n* = 9 GFP+Cre basket neurons for bouton density and terminal branching; *n* = 4 GFP Ctrl vs *n* = 5 GFP+Cre basket neurons for bouton size analysis). ***E***, Representative immunofluorescence for eGFP and NeuN in *Pvalb*^Cre^, RCE, *Sox6*^fl/±^ at P28, and bar blot shows eGFP+ bouton size around pyramidal neurons (*n* = 3 mice per genotype; bouton size value is the average of all boutons surrounding 15–30 cells per animal; unpaired *t* test, *p* = 0.033; boutons per soma, *p* = 0.78). ***F***, Whole-cell patch-clamp recordings of cortical Pvalb^+^ neurons and pyramidal neurons from S1 acute slices from control and Sox6-cKO mice. Paired recordings of Pvalb^+^ neuron evoked postsynaptic currents in neighboring pyramidal neuron. Presynaptic Pvalb^+^ neuron (in current-clamp) and postsynaptic evoked currents in pyramidal neuron (voltage-clamp recording with high-chloride intracellular solution). ***G***, Percentage of failure rate of Pvalb^+^ neurons' output after one AP (*n* = 3 cells from *n* = 3 mice per condition; unpaired *t* test, *p* = 0.007). ***H***, Percentage of average failure rate during five APs at 10 Hz (right; unpaired *t* test, *p* = 0.042). ***I***, Summary of PPR (IPSC_2_/IPSC_1_) for the first pair of APs (*n* = 3 mice per condition; unpaired *t* test, *p* = 0.008). ***J***, PPR during spike trains of five APs [two-way ANOVA, repeated measures, row factor (genotype) *p* = 0.025; and column factor (IPSC_N_) = *p* < 0.001]. EP, equivalent postnatal day; PPR, paired-pulse ratio; Py, pyramidal neuron; Vh, voltage holding; *p < 0.05, **p < 0.01. Error bars, SEM. Scale bar: 5 µm.

Briefly, organotypic cultures were prepared from P3 to P5 *Sox6*^fl/fl^ mice. Subsequently, we transfected cells with P_G67_ plasmid alone to drive the expression of GFP (Control Pvalb^+^ neurons) or P_G67_ together with Cre (Sox6-cKO Pvalb^+^ neurons). Using this approach, we could conditionally knock-out *Sox6* in single Pvalb^+^ basket cells at different developmental windows, in an otherwise wild-type background (Fig. 2). Transfections were performed either between equivalent P (EP)10 (P5 plus 5 d *in vitro)* and analyzed at EP18 or from EP26 to P34 (Fig. 2*A*,*B*).

We have previously shown that the large majority of GFP-labeled boutons in these experimental conditions represent presynaptic terminals (Chattopadhyaya et al., 2004; Wu et al., 2012). *Sox6* removal from EP10 to EP18, a phase of active axonal growth (Chattopadhyaya et al., 2004), did not disrupt overall bouton density and axon branching around pyramidal cell somata but led to a reduction in the size of individual boutons (Fig. 2*C*). Removing *Sox6* at EP26, a time point when the axonal structure of basket cells *in vitro* is mature and much more stable (Chattopadhyaya et al., 2004; Baho and Di Cristo, 2012; Baho et al., 2019), also led to decreased bouton size (Fig. 2*D*). In addition, while wild-type EP34 Pvalb^+^ basket cells had significantly larger bouton size than EP18 Pvalb^+^ basket cells (one-way ANOVA followed by *post hoc* Tukey's multiple comparison, *p* = 0.0011), the same comparison between mutant cells showed no significant difference (*p* = 0.0629). Similarly, we found no significant differences in bouton size between EP18 wild-type Pvalb^+^ and EP34 Sox6-cKO Pvalb^+^ basket cells (*p* = 0.9222). Altogether these data suggest that postnatal loss of *Sox6* in individual Pvalb^+^ basket cells impaired synaptic bouton growth.

To investigate whether the observed effects were recapitulated *in vivo*, we analyzed GFP+ boutons in S1 of P28 *Pvalb*^Cre^:RCE:*Sox6*^fl/±^ mice. This analysis revealed a similar reduction in bouton size in Sox6-cKO mice compared with littermate controls (unpaired *t* test, *p* = 0.033; Fig. 2*E*) and no significant effect in number of bouton per soma (unpaired *t* test, *p* = 0.78; Fig. 2*E*). Importantly, to investigate whether the structural phenotype had a functional correlate, we performed paired recordings of LII/III Pvalb^+^ cell and neighboring pyramidal neurons in P16–P28 S1 acute slices of *Pvalb*^Cre^:RCE:*Sox6*^fl/±^ mice (Fig. 2*F*). As previously shown (Goldberg et al., 2011), we confirmed the high release probability and low failure rates of Pvalb^+^ synapses in controls (Fig. 2*G*). Conversely, in paired recordings from Sox6-cKO mice we observed increased failure rate of evoked IPSCs (Fig. 2G, unpaired *t* test, *p* = 0.007, H, *p* = 0.042) and higher paired-pulse ratio [Fig. 2*I*, unpaired *t* test, *p* = 0.027, *J*, two-way ANOVA, repeated measures, row factor (genotype) *p* = 0.025; and column factor (IPSC_N_) = *p* < 0.001], suggestive of a presynaptic effect and indicative of lower release probability. Overall, these data suggest that *Sox6* regulates synaptic output in postnatal Pvalb^+^ neurons.

### *In vivo* adult *Sox6* removal affects *pvalb*^+^ neuron axonal bouton size

**Figure 3.**
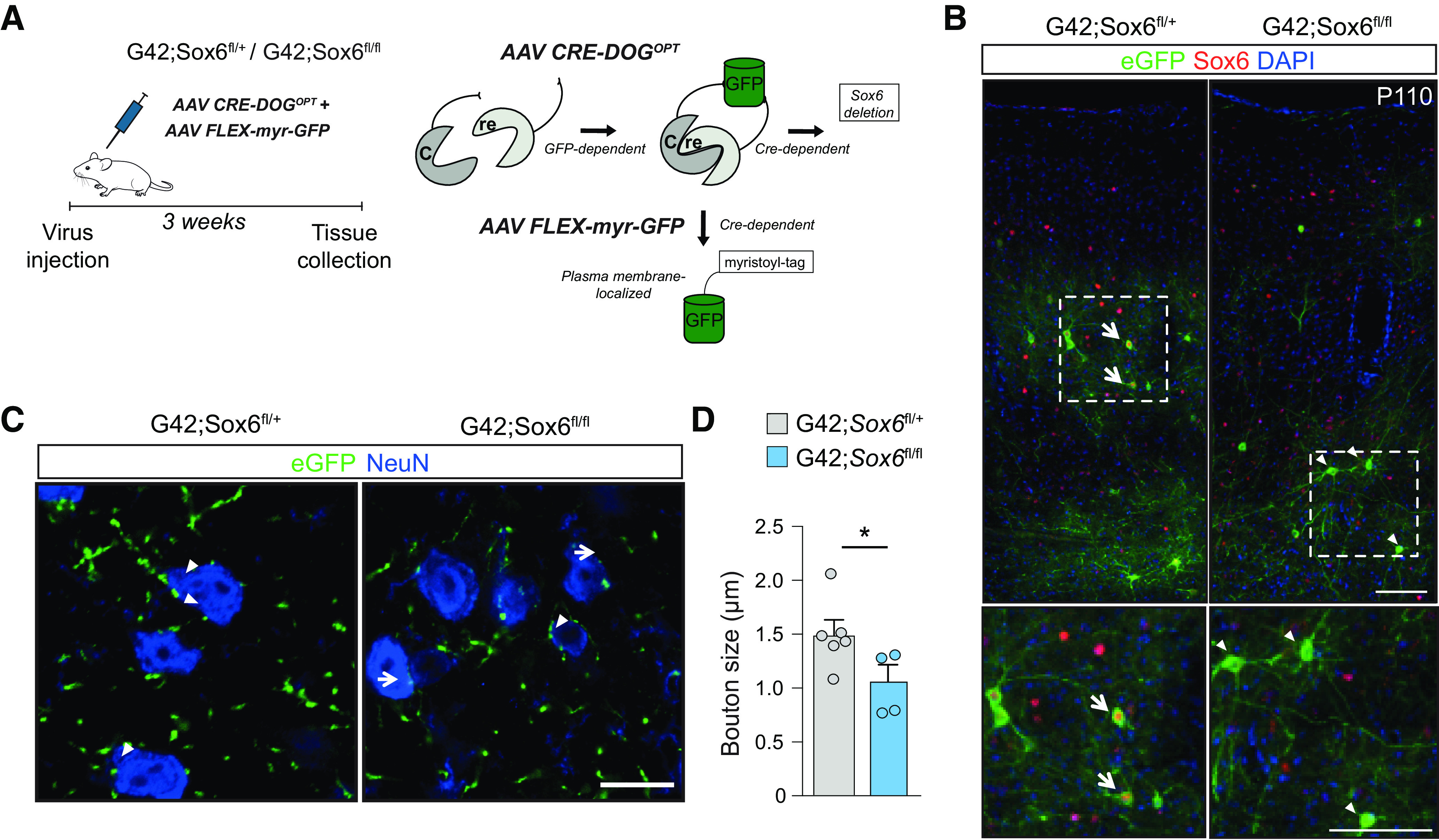
Sox6 loss in adult Pvalb^+^ neurons is sufficient to decrease Pvalb^+^ neuron bouton size. ***A***, Left, Schematic of adult removal of Sox6 in Pvalb neurons. Right, Schematic of viral approach. ***B***, Immunofluorescence of S1 cortical sections (injection site). Left, In control animals (G42; *Sox6*^fl/+^ *n* = 4 mice), transfected cells still express Sox6 (arrows). Right, In G42;*Sox6*^fl/fl^ mice (*n* = 5 mice), Sox6 expression is efficiently ablated in transfected cells (arrowheads). Scale bar: 50 µm. ***C***, Representative immunofluorescence for eGFP and NeuN on injection site three weeks after transfection, as in ***B***. Scale bar: 20 µm. ***D***, Bar plots show bouton size (eGFP^+^) surrounding pyramidal neurons' soma (NeuN^+^; control *n* = 6 and Sox6-cKO *n* = 4 mice; individual values are averages from 50 to 100 boutons per mouse; unpaired *t* test, *p* = 0.048); *p < 0.05. Error bars, SEM.

Next, we wanted to investigate whether Sox6 regulates synaptic stability in Pvalb^+^ neurons after adolescence, since around 75% of Pvalb^+^ neurons in S1 still express Sox6 at P90 (Fig. 1*A*,*B*). In order to remove *Sox6* in the adult mouse cortex, we used a combination of three viral vectors: two adeno-associated viruses (AAVs) carrying Cre-recombinase dependent on GFP (Cre-DOG; in which Cre is assembled depending on the presence of GFP) and an AAV carrying Flex myr-GFP (Cre-dependent expression of myristoylation-GFP, targeting GFP to the membrane). These viruses were unilaterally injected into three- to four-month-old *Sox6*^fl/fl,+^ mice crossed onto G42 (G42:*Sox6*^fl/fl,+^) which is a GAD67-eGFP transgenic mouse line labeling a subset of Pvalb^+^ neurons (Chattopadhyaya et al., 2004; Fig. 3*A*). The effect of the viruses was restricted to cells expressing eGFP and follows a two-step process: first to excise *Sox6* (Cre-DOG) but also to drastically enhance the eGFP signal allowing for visualization of synaptic boutons (myr-GFP). Twenty-one days after transfection, we confirmed with immunohistochemistry that eGFP^+^ cells within the injection site did not express Sox6 in the G42:Sox6^fl/fl^ mice (Fig. 3*B*), leaving Sox6 expression unaffected in non-transfected neurons and in neurons from controls (G42:*Sox6*^fl/+^). We then quantified the size of eGFP^+^ boutons surrounding NeuN^+^ cell somata, revealing a significant reduction in bouton size after Sox6 loss (unpaired *t* test, *p* = 0.048; Fig. 3*C*,*D*), suggesting that *Sox6* plays an active, cell-autonomous role in regulating synaptic bouton size also in adult cortex.

### Sox6 modulates Pvalb^+^ basket cell innervation by regulating TrkB expression

One of the strongest known modulators of GABAergic synapses' development, maturation and adult regulation is the neurotrophin BDNF, which acts by binding TrkB. In particular, TrkB ablation in Pvalb^+^ neurons in the neocortex and hippocampus leads to synaptic deficits (Ohba et al., 2005; Zheng et al., 2011; Xenos et al., 2018) and to fewer GABAergic boutons when removed in GABAergic neurons in the cerebellum (Rico et al., 2002). Scaling of GABAergic synapse strength is also regulated by BDNF (Swanwick et al., 2006; Hong et al., 2008), with loss of activity-dependent BDNF release causing impaired Pvalb^+^ cell maturation and function (Jiao et al., 2011). Furthermore, adult loss of TrkB in cortical Pvalb^+^ neurons leads to decreased inhibition onto pyramidal neurons and abnormal cortical network activity (Tan et al., 2018; Guyon et al., 2021).

**Figure 4.**
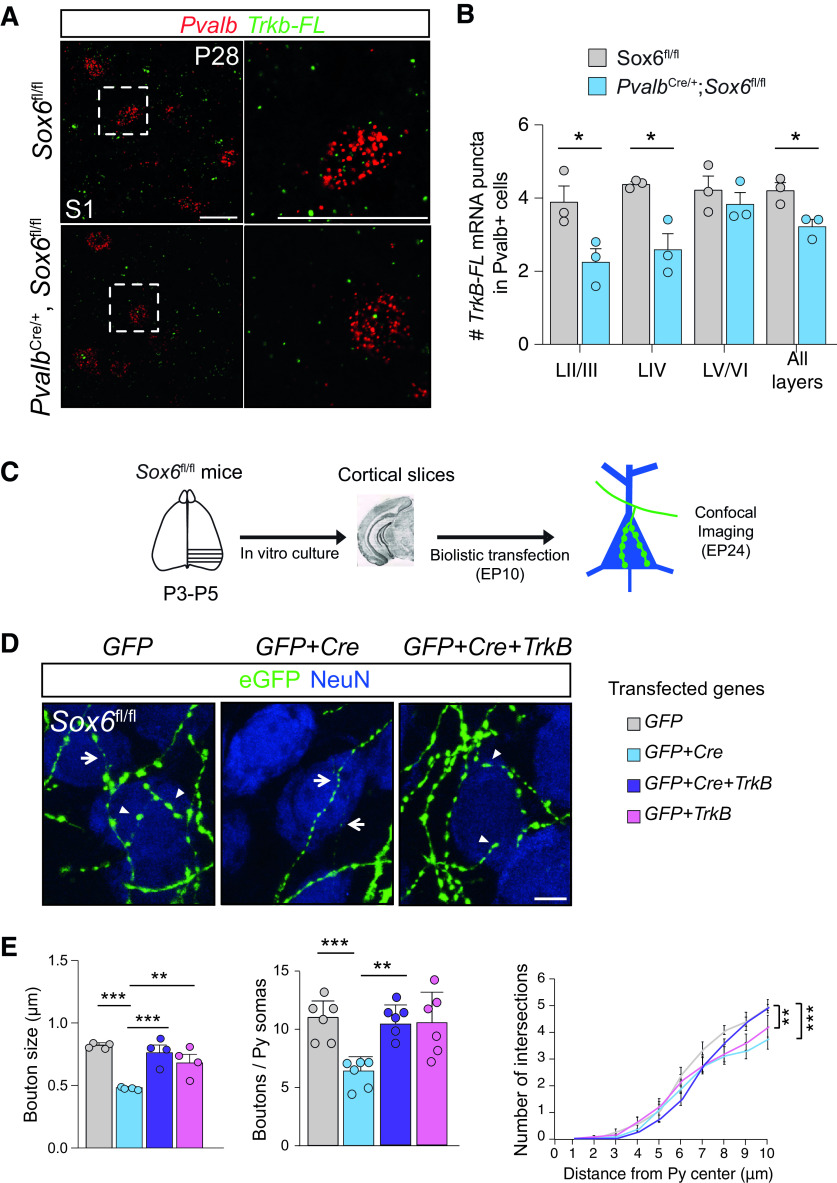
TrkB mediates Sox6's role in the maturation of Pvalb^+^ neuron synaptic innervation. ***A***, *In situ* hybridization for *Pvalb* and *TrkB-full-length* (TrkB-FL) in S1 cortical sections of P28 mice. Scale bar: 20 µm. ***B***, Bar plots display number of *TrkB-FL* mRNA puncta in *Pvalb*+ cells (*Pvalb* used as mask) in control (*Sox6*^fl/fl^) and Sox6-mutant (*Pvalb*^Cre^, *Sox6*^fl/fl^) mice (*n* = 3 mice per genotype; average value from 50 to 100 *Pvalb*+ cells per animal; unpaired *t* test, LII/III, *p* = 0.042; LIV, *p* = 0.014; LV/VI, *p* = 0.46; all layers, *p* = 0.02). ***C***, Scheme of organotypic slice preparation for analysis of basket cell perisomatic innervation from EP10 to EP24. ***D***, Representative immunofluorescence of EP10–EP24 organotypic slices from Sox6^fl/fl^ mice. Single basket cells were transfected with either only P_G67_-*GFP* plasmid (left, control), or P_G67_-*GFP*+*Cre* plasmid (center, single-cell Sox6−/−), or P_G67_-*GFP*+*Cre*+*TrkB* (right, rescue) Scale bar: 5 µm. ***E***, Bar plots show bouton size (one-way ANOVA with *post hoc* Tukey's test P_G67_-*GFP* vs P_G67_-*GFP*+*Cre p* = 0.0002*;* P_G67_-*GFP*+*Cre* vs P_G67_-*GFP*+*Cre*+*TrkB p* = 0.0162; P_G67_-*GFP*+*Cre* vs P_G67_-*GFP*+*TrkB p* = 0.0013; P_G67_-*GFP* vs P_G67_-*GFP*+*TrkB p* = 0.7; *n* = 4 basket cells for all conditions), bouton density (one-way ANOVA with *post hoc* Tukey's test P_G67_-*GFP* vs P_G67_-*GFP*+*Cre p* = 0.0016*;* P_G67_-*GFP*+*Cre* vs P_G67_-*GFP*+*Cre*+*TrkB p* = 0.0103; P_G67_-*GFP*+*Cre* vs P_G67_-*GFP*+*TrkB p* = 0.0094; P_G67_-*GFP* vs P_G67_-*GFP*+*TrkB p* = 0.8665; *n* = 6 basket cells for all conditions), and terminal branching (one-way ANOVA with *post hoc* Tukey's test at 9 µm P_G67_-*GFP* vs P_G67_-*GFP*+*Cre p* = 0.0026*;* P_G67_-*GFP*+*Cre* vs P_G67_-*GFP*+*Cre*+*TrkB p* = 0.0124; *n* = 6 basket cells for all conditions). EP, equivalent postnatal day; Py, pyramidal neuron; **p < 0.01, ***p < 0.001. Error bars, SEM.

We therefore hypothesized that TrkB acts downstream of Sox6 and mediates synaptic maintenance in Pvalb^+^ neurons. Consistent with our hypothesis, *in situ* hybridization for *Pvalb* and full-length *TrkB* (gene *Ntrk2*) in P28 *Pvalb*^Cre^: *Sox6*^fl/fl^ compared with Cre-negative littermates (*Sox6*^fl/fl^) revealed that loss of Sox6 decreases expression of *TrkB-FL* in Pvalb^+^ neurons, as shown by the reduced number of *TrkB-FL* puncta in *Pvalb*+ cells (unpaired *t* test: # *TrkB-FL* puncta in *Pvalb*+ cells: LII/III, *p* = 0.0412; LIV, *p* = 0.014; LV/VI, *p* = 0.46; all layers, *p* = 0.023; Fig. 4*A*,*B*). In accordance with unaffected Pvalb protein expression (Fig. 1*F*,*H*), Sox6 ablation did not affect Pvalb mRNA levels (unpaired *t* test: mean intensity *Pvalb*: LII/III, *p* = 0.92; LIV, *p* = 0.30; LV/VI, *p* = 0.63; all layers, *p* = 0.38).

In order to investigate whether TrkB mediates Sox6's action on refinement of Pvalb^+^ neurons' synaptic connectivity, we prepared organotypic slices from *Sox6*^fl/fl^ mice and transfected them from EP10 to EP24 using four different experimental conditions (plasmid combinations): wild-type Pvalb^+^ basket cells (transfected with only GFP), Sox6 deficient Pvalb^+^ basket cells (transfected with *GFP* + *Cre*), Sox6 deficient Pvalb^+^ basket cells re-expressing TrkB (transfected with *GFP* + *Cre* + *TrkB* cDNA) and wild-type Pvalb^+^ basket cell over-expressing TrkB (transfected with *GFP* + *TrkB* cDNA (Fig. 4*C–E*).

Notably, re-expression of *TrkB* in Pvalb^+^ basket cells lacking *Sox6* significantly rescued perisomatic bouton size (*GFP* + *Cre* + *Trkb*: 0.69 ± 0.06 µm one-way ANOVA *post hoc* Tukey's multiple comparisons, compared with *GFP* + *Cre* adjusted *p* = 0.0162; compared with *GFP* adjusted *p* = 0.0987; Fig. 4*E*). Interestingly, in contrast to the short-term effect of Sox6 loss which only affects bouton size (EP10–EP18; Fig. 2*C*), long-term loss of Sox6 also affects overall bouton density and axonal branching around pyramidal cell somata (EP10–EP24; Fig. 4*E*), possibly because of pruning of small non-functional synaptic boutons over time. Re-expression of *TrkB* in Pvalb^+^ basket cells lacking *Sox6* also rescued perisomatic bouton density [*GFP* + *Cre* + *Trkb*: 9.6 ± 0.5 boutons/soma one-way ANOVA *post hoc* Tukey's multiple comparisons, compared with *GFP* + *Cre* (5.92 ± 0.5 boutons/soma) adjusted *p* = 0.01; compared with *GFP* (10.51 ± 0.67 boutons/soma) adjusted *p* = 0.84; Fig. 4*E*]. Altogether, these data strongly suggest a *Sox6*-mediated cell-autonomous regulatory role on TrkB expression, which in turn mediates fine-tuning of Pvalb^+^ cell innervation during postnatal development.

### Sox6 expression is upregulated in primary motor cortex (M1) Pvalb^+^ neurons following increased locomotor activity

**Figure 5.**
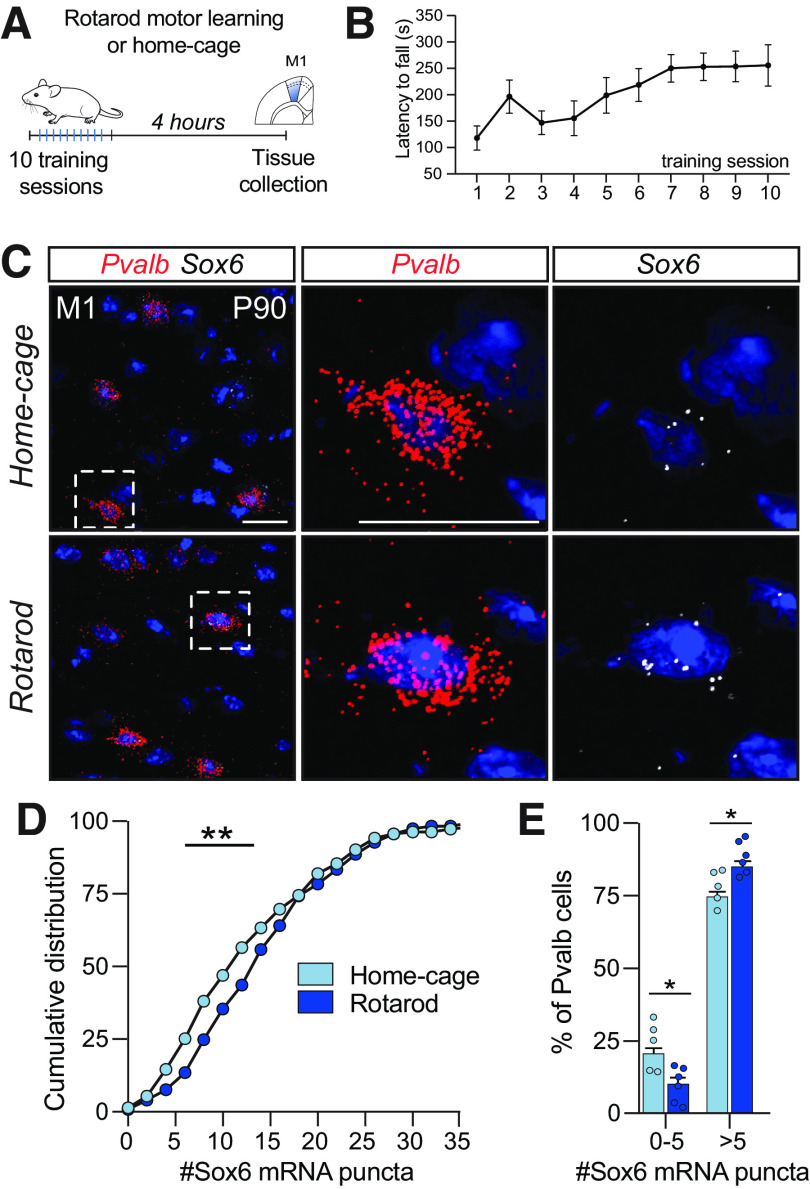
Sox6 expression can be regulated in adult cortical Pvalb^+^ neurons. ***A***, Schematic of experimental design in which adult mice experienced ten sessions of an accelerating rotarod and brains were collected 4 h after last session. Home-cage littermates were used as controls. ***B***, Plot showing increase in latency to fall throughout sessions (*n* = 6 mice). ***C***, Representative images of *in situ* hybridization for Pvalb and Sox6 of M1. ***D***, Cumulative distribution of fractions of Pvalb^+^ neurons according to numbers of Sox6 puncta expressed (Kolmogorov–Smirnov test; *p* = 0.0078). ***E***, Bar plots show that changes in percent of Pvalb neurons expressing 0–5 or >5 Sox6 mRNA puncta (*n* = 5–6 mice per condition; values correspond to the average of 50–100 Pvalb^+^ cells per animal; unpaired *t* test, *p* = 0.021); M1, primary motor cortex; *p < 0.05, **p < 0.01. Error bars, SEM. Scale bar: 5 µm.

Interestingly, the *Sox6* gene contains a synaptic activity-responsive element (SARE), a regulatory enhancer element originally discovered in the immediate early gene *Arc* (Kawashima et al., 2009) that predicts transcriptional changes in response to neuronal activation (Rodriguez-Tornos et al., 2013; Pulimood et al., 2017). Therefore, to test the hypothesis that *Sox6* expression in cortical Pvalb^+^ cells can be modulated by increased network activity, we moved to the M1, a cortical region engaged during motor learning (Kleim et al., 1996, 1998; Beloozerova et al., 2003; Costa et al., 2004; Hosp et al., 2013; Cao et al., 2015; Andreska et al., 2020). We used the accelerating rotarod task as a means of enforcing locomotor activity and thus triggering increased neuronal firing in M1 (Costa et al., 2004), which augments expression of activity-regulated genes in both pyramidal neurons (Cao et al., 2015; Hirata et al., 2016) and in Pvalb^+^ cells (Arango-Lievano et al., 2019). In this task, mice were placed on a rotating rod under continuous acceleration for 10 consecutive trials (6 min each). Latency to fall was tracked to evaluate improvement in balance and motor coordination (Fig. 5*A*,*B*).

Activity-driven changes in gene expression vary across different neuronal subtypes (Mardinly et al., 2016) and different patterns and durations of stimuli can induce temporally distinct genetic programs (Tyssowski et al., 2018). To account for that, we collected the brains from trained and home-cage littermate controls 4 h after the last rotarod session, a time point in which both rapid and delayed waves of activity-modulated gene expression remain elevated (Tyssowski et al., 2018). *In situ* hybridization for *Pvalb* and *Sox6* in M1 revealed that Pvalb^+^ neurons from trained mice expressed a higher number of *Sox6* puncta than controls (Fig. 5*C*,*D*, Kolmogorov–Smirnov test; *p* = 0.0078, *E*, unpaired *t* test, *p* = 0.021), thus suggesting that Sox6 expression in cortical *Pvalb*^+^ can indeed be modulated by activity.

## Discussion

Here, we showed that Sox6 is an activity-modulated transcription factor constitutively expressed in cortical Pvalb^+^ neurons and identified it as a key-player in shaping cortical inhibitory synaptic output via control of TrkB expression, from postnatal development and into adulthood.

From the second postnatal week, Pvalb^+^ neurons go through a gradual shift in their molecular and electrophysiological maturation (Okaty et al., 2009; Goldberg et al., 2011). In parallel, their axonal arbor increases in complexity and contacts a progressively larger number of postsynaptic targets with large boutons clustered around the somata and proximal dendrites of the postsynaptic neurons (Chattopadhyaya et al., 2004; Favuzzi et al., 2017). This maturational process plateaus only by the end of the fourth postnatal week (Chattopadhyaya et al., 2004). While embryonic expression of Sox6 is essential for layer allocation and maturation of intrinsic electrophysiological properties of Pvalb^+^ neurons (Batista-Brito et al., 2009), here we show that postnatally the role of Sox6 shifts toward regulating synapse maturation and maintenance, suggesting that Sox6 plays distinct yet essential roles in different developmental processes of Pvalb^+^ neurons. Furthermore, our data suggest that, while postnatal expression of Sox6 is dispensable for certain features of Pvalb^+^ neurons' maturation, it is required for synaptic maturation and synaptic stability, therefore indicating that distinct maturational and cellular processes can be independently controlled by different molecular programs. Accordingly, Sox6 is a transcription factor that does not regulate gene expression by direct biding to DNA regulatory sequences (Connor et al., 1994). Instead Sox6 acts together with cell-type specific partner proteins, which consequently delineate the specificity of genes to be regulated in distinct cell types and timepoints (Kamachi et al., 2000; Stolt et al., 2006; Hagiwara, 2011). To our knowledge, it remains unknown which specific partners Sox6 recruits in Pvalb^+^ neurons, but other transcriptions factors have been shown to partner with Sox6, such as Sox5 and CtBP2, in non-neural tissue (Lefebvre et al., 1998; Murakami et al., 2001; Hagiwara, 2011).

Our findings that Sox6 levels in Pvalb^+^ neurons is modulated by increased locomotor activity suggests that it could be an important element in sensing changes in activity and shaping cortical inhibitory synaptic output via control of TrkB expression. In cortical pyramidal neurons, TrkB expression is regulated through activity-dependent activation of the *Ntrk2* P2 promoter by calcium responsive elements down-stream of calcium influx and CREB activation (Kingsbury et al., 2003; Deogracias et al., 2004). While less is known about which factors control TrkB expression in Pvalb^+^, BDNF-TrkB signaling regulates GABA synthesis in cortical interneurons in a CREB-dependent manner (Sánchez-Huertas and Rico, 2011), suggesting a regulatory pathway also based on activity levels. Accordingly, BDNF-TrkB signaling is closely linked to the fine-tuning and facilitation of inhibitory drive to excitatory neurons (Colino-Oliveira et al., 2016; Gu et al., 2018; Porcher et al., 2018). For example, activity-dependent expression of *Npas4* in pyramidal cells increases BDNF release, which acts on presynaptic TrkB receptors on Pvalb^+^ neurons to recruit inhibition (Bloodgood et al., 2013; Spiegel et al., 2014). Similar to the activity-dependent mechanisms regulating BDNF release, TrkB activation and expression in excitatory neurons (Dragunow et al., 1993, 1997), our findings that Sox6 regulates TrkB expression suggest it to be an important presynaptic regulator in Pvalb^+^ neurons.

In particular, Pvalb^+^ neurons fine-tune the strength of somatic inhibition they provide and are especially prone to short-term potentiation, based on the activity of the individual pyramidal cells (Lourenço et al., 2014; Xue et al., 2014). Furthermore, TrkB expression in Pvalb^+^ neurons is necessary for inducing antidepressant effects in adult mice (Lesnikova et al., 2021) while motor learning induces transient increase in Pvalb^+^ synaptic bouton density across days (Chen et al., 2015). Therefore, it is possible that Sox6's role in regulating TrkB mRNA expression covers a timescale that requires transcription and that it is perhaps less responsive to short-term changes in excitability, but on the contrary confers potentially more long-lasting effects (Dragunow et al., 1993, 1997; Lin et al., 2018).

## References

[B1] Amegandjin CA, Choudhury M, Jadhav V, Carriço JN, Quintal A, Berryer M, Snapyan M, Chattopadhyaya B, Saghatelyan A, Di Cristo G (2021) Sensitive period for rescuing parvalbumin interneurons connectivity and social behavior deficits caused by TSC1 loss. Nat Commun 12:3653. 34135323 10.1038/s41467-021-23939-7 PMC8209106

[B2] Anderson SA, Eisenstat DD, Shi L, Rubenstein JL (1997) Interneuron migration from basal forebrain to neocortex: dependence on Dlx genes. Science 278:474–476. 10.1126/science.278.5337.474 9334308

[B3] Anderson SA, Marin O, Horn C, Jennings K, Rubenstein JL (2001) Distinct cortical migrations from the medial and lateral ganglionic eminences. Development 128:353–363. 11152634 10.1242/dev.128.3.353

[B4] Andreska T, Rauskolb S, Schukraft N, Lüningschrör P, Sasi M, Signoret-Genest J, Behringer M, Blum R, Sauer M, Tovote P, Sendtner M (2020) Induction of BDNF expression in layer II/III and layer V neurons of the motor cortex is essential for motor learning. J Neurosci 40:6289–6308. 10.1523/JNEUROSCI.0288-20.2020 32651187 PMC7424868

[B5] Arango-Lievano M, Borie Am, Dromard Y, Murat M, Desarmenien MG, Garabedian MJ, Jeanneteau F (2019) Persistence of learning-induced synapses depends on neurotrophic priming of glucocorticoid receptors. Proc Natl Acad Sci USA 116:13097–13106. 31182610 10.1073/pnas.1903203116 PMC6601006

[B6] Baho E, Chattopadhyaya B, Lavertu-Jolin M, Mazziotti R, Awad PN, Chehrazi P, Groleau M, Jahannault-Talignani C, Vaucher E, Ango F, Pizzorusso T, Baroncelli L, Di Cristo G (2019) p75 neurotrophin receptor activation regulates the timing of the maturation of cortical parvalbumin interneuron connectivity and promotes juvenile-like plasticity in adult visual cortex. J Neurosci 39:4489–4510. 10.1523/JNEUROSCI.2881-18.2019 30936240 PMC6554620

[B7] Baho E, Di Cristo G (2012) Neural activity and neurotransmission regulate the maturation of the innervation field of cortical GABAergic interneurons in an age-dependent manner. J Neurosci 32:911–8. 22262889 10.1523/JNEUROSCI.4352-11.2012 PMC6621145

[B8] Banerjee SB, Gutzeit VA, Baman J, Aoued HS, Doshi NK, Liu RC, Ressler KJ (2017) Perineuronal nets in the adult sensory cortex are necessary for fear learning. Neuron 95:169–179.e3. 28648500 10.1016/j.neuron.2017.06.007 PMC5548423

[B9] Bartolini G, Ciceri G, Marín O (2013) Integration of GABAergic interneurons into cortical cell assemblies: lessons from embryos and adults. Neuron 79:849–864. 10.1016/j.neuron.2013.08.014 24012001

[B10] Batista-Brito R, Rossignol E, Hjerling-Leffler J, Denaxa M, Wegner M, Lefebvre V, Pachnis V, Fishell G (2009) The cell-intrinsic requirement of Sox6 for cortical interneuron development. Neuron 63:466–481. 19709629 10.1016/j.neuron.2009.08.005 PMC2773208

[B11] Beloozerova IN, Sirota MG, Swadlow HA (2003) Activity of different classes of neurons of the motor cortex during locomotion. J Neurosci 23:1087–1097. 12574439 10.1523/JNEUROSCI.23-03-01087.2003 PMC6741911

[B12] Bloodgood BL, Sharma N, Browne HA, Trepman AZ, Greenberg ME (2013) The activity-dependent transcription factor NPAS4 regulates domain-specific inhibition. Nature 503:121–125. 24201284 10.1038/nature12743 PMC4169177

[B13] Cao VY, Ye Y, Mastwal S, Ren M, Coon M, Liu Q, Costa RM, Wang KH (2015) Motor learning consolidates Arc-expressing neuronal ensembles in secondary motor cortex. Neuron 86:1385–1392. 26051420 10.1016/j.neuron.2015.05.022 PMC4474764

[B14] Chattopadhyaya B, Di Cristo G, Higashiyama H, Knott GW, Kuhlman SJ, Welker E, Huang ZJ (2004) Experience and activity-dependent maturation of perisomatic GABAergic innervation in primary visual cortex during a postnatal critical period. J Neurosci 24:9598–9611. 15509747 10.1523/JNEUROSCI.1851-04.2004 PMC6730138

[B15] Chattopadhyaya B, Di Cristo G, Wu CZ, Knott G, Kuhlman S, Fu Y, Palmiter RD, Huang ZJ (2007) GAD67-mediated GABA synthesis and signaling regulate inhibitory synaptic innervation in the visual cortex. Neuron 54:889–903. 17582330 10.1016/j.neuron.2007.05.015 PMC2077924

[B16] Chattopadhyaya B, Baho E, Huang ZJ, Schachner M, Di Cristo G (2013) Neural cell adhesion molecule-mediated Fyn activation promotes GABAergic synapse maturation in postnatal mouse cortex. J Neurosci 33:5957–5968. 23554477 10.1523/JNEUROSCI.1306-12.2013 PMC6618917

[B17] Chen SX, Kim AN, Peters AJ, Komiyama T (2015) Subtype-specific plasticity of inhibitory circuits in motor cortex during motor learning. Nat Neurosci 18:1109–1115. 26098758 10.1038/nn.4049 PMC4519436

[B18] Colino-Oliveira M, Rombo DM, Dias RB, Ribeiro JA, Sebastião AM (2016) BDNF-induced presynaptic facilitation of GABAergic transmission in the hippocampus of young adults is dependent of TrkB and adenosine A2A receptors. Purinergic Signal 12:283–294. 10.1007/s11302-016-9502-y 26897393 PMC4854840

[B19] Connor F, Cary PD, Read CM, Preston NS, Driscoll PC, Denny P, Crane-Robinson C, Ashworth A (1994) DNA binding and bending properties of the post-meiotically expressed Sry-related protein Sox-5. Nucleic Acids Res 22:3339–3346. 8078769 10.1093/nar/22.16.3339 PMC523727

[B20] Costa RM, Cohen D, Nicolelis MA (2004) Differential corticostriatal plasticity during fast and slow motor skill learning in mice. Curr Biol 14:1124–1134. 15242609 10.1016/j.cub.2004.06.053

[B21] Dehorter N, Ciceri G, Bartolini G, Lim L, del Pino I, Marín O (2015) Tuning of fast-spiking interneuron properties by an activity-dependent transcriptional switch. Science 349:1216–1220. 26359400 10.1126/science.aab3415 PMC4702376

[B22] Deogracias R, Espliguero G, Iglesias T, Rodríguez-Peña A (2004) Expression of the neurotrophin receptor trkB is regulated by the cAMP/CREB pathway in neurons. Mol Cell Neurosci 26:470–480. 15234351 10.1016/j.mcn.2004.03.007

[B23] Di Cristo G, Chattopadhyaya B, Kuhlman SJ, Fu Y, Belanger MC, Wu CZ, Rutishauser U, Maffei L, Huang ZJ (2007) Activity-dependent PSA expression regulates inhibitory maturation and onset of critical period plasticity. Nat Neurosci 10:1569–1577. 18026099 10.1038/nn2008

[B24] Dragunow M, Beilharz E, Mason B, Lawlor P, Abraham W, Gluckman P (1993) Brain-derived neurotrophic factor expression after long-term potentiation. Neurosci Lett 160:232–236. 8247360 10.1016/0304-3940(93)90420-p

[B25] Dragunow M, Hughes P, Mason-Parker SE, Lawlor P, Abraham WC (1997) TrkB expression in dentate granule cells is associated with a late phase of long-term potentiation. Brain Res Mol Brain Res 46:274–280. 9191102 10.1016/s0169-328x(97)00021-1

[B26] Dumitriu B, Dy P, Smits P, Lefebvre V (2006) Generation of mice harboring a Sox6 conditional null allele. Genesis 44:219–224. 16652367 10.1002/dvg.20210

[B27] Fan Y, Fricker D, Brager DH, Chen X, Lu HC, Chitwood RA, Johnston D (2005) Activity-dependent decrease of excitability in rat hippocampal neurons through increases in I(h). Nat Neurosci 8:1542–1551. 16234810 10.1038/nn1568

[B28] Favuzzi E, Marques-Smith A, Deogracias R, Winterflood CM, Sanchez-Aguilera A, Mantoan L, Maeso P, Fernandes C, Ewers H, Rico B (2017) Activity-dependent gating of parvalbumin interneuron function by the perineuronal net protein brevican. Neuron 95:639–655.e10. 28712654 10.1016/j.neuron.2017.06.028

[B29] Goldberg EM, Jeong HY, Kruglikov I, Tremblay R, Lazarenko RM, Rudy B (2011) Rapid developmental maturation of neocortical FS cell intrinsic excitability. Cereb Cortex 21:666–682. 20705896 10.1093/cercor/bhq138 PMC3041012

[B30] Gu F, Parada I, Yang T, Longo FM, Prince DA (2018) Partial TrkB receptor activation suppresses cortical epileptogenesis through actions on parvalbumin interneurons. Neurobiol Dis 113:45–58. 29408225 10.1016/j.nbd.2018.01.018

[B31] Guyon N, Zacharias LR, Van Lunteren JA, Immenschuh J, Fuzik J, Märtin A, Xuan Y, Zilberter M, Kim H, Meletis K, Lopes-Aguiar C, Carlén M (2021) Adult trkB signaling in parvalbumin interneurons is essential to prefrontal network dynamics. J Neurosci 41:3120–3141. 33593856 10.1523/JNEUROSCI.1848-20.2021 PMC8026352

[B32] Hagiwara N (2011) Sox6, jack of all trades: a versatile regulatory protein in vertebrate development. Dev Dyn 240:1311–1321. 10.1002/dvdy.22639 21495113 PMC3092843

[B33] Hippenmeyer S, Vrieseling E, Sigrist M, Portmann T, Laengle C, Ladle Dr, Arber S (2005) A developmental switch in the response of DRG neurons to ETS transcription factor signaling. PLoS Biol 3:e159. 15836427 10.1371/journal.pbio.0030159 PMC1084331

[B34] Hirata H, Takahashi A, Shimoda Y, Koide T (2016) Caspr3-deficient mice exhibit low motor learning during the early phase of the accelerated rotarod task. PLoS One 11:e0147887. 26807827 10.1371/journal.pone.0147887 PMC4726695

[B35] Hong EJ, Mccord AE, Greenberg ME (2008) A biological function for the neuronal activity-dependent component of Bdnf transcription in the development of cortical inhibition. Neuron 60:610–624. 19038219 10.1016/j.neuron.2008.09.024 PMC2873221

[B36] Hosp JA, Mann S, Wegenast-Braun BM, Calhoun ME, Luft AR (2013) Region and task-specific activation of Arc in primary motor cortex of rats following motor skill learning. Neuroscience 250:557–564. 10.1016/j.neuroscience.2013.06.060 23876329

[B37] Hu H, Gan J, Jonas P (2014) GABAergic interneurons: from cellular design to microcircuit function. Science 345:1255263. 25082707 10.1126/science.1255263

[B38] Inan M, Welagen J, Anderson SA (2012) Spatial and temporal bias in the mitotic origins of somatostatin- and parvalbumin-expressing interneuron subgroups and the chandelier subtype in the medial ganglionic eminence. Cereb Cortex 22:820–827. 10.1093/cercor/bhr148 21693785 PMC3450921

[B39] Jiao Y, Zhang Z, Zhang C, Wang X, Sakata K, Lu B, Sun QQ (2011) A key mechanism underlying sensory experience-dependent maturation of neocortical GABAergic circuits in vivo. Proc Natl Acad Sci USA 108:12131–12136. 21730187 10.1073/pnas.1105296108 PMC3141955

[B40] Kamachi Y, Uchikawa M, Kondoh H (2000) Pairing SOX off: with partners in the regulation of embryonic development. Trends Genet 16:182–187. 10729834 10.1016/s0168-9525(99)01955-1

[B41] Kawashima T, Okuno H, Nonaka M, Adachi-Morishima A, Kyo N, Okamura M, Takemoto-Kimura S, Worley PF, Bito H (2009) Synaptic activity-responsive element in the Arc/Arg3.1 promoter essential for synapse-to-nucleus signaling in activated neurons. Proc Natl Acad Sci USA 106:316–321. 19116276 10.1073/pnas.0806518106 PMC2629236

[B42] Kingsbury TJ, Murray PD, Bambrick LL, Krueger BK (2003) Ca(2+)-dependent regulation of TrkB expression in neurons. J Biol Chem 278:40744–40748. 12900419 10.1074/jbc.M303082200

[B43] Kleim JA, Lussnig E, Schwarz ER, Comery TA, Greenough WT (1996) Synaptogenesis and FOS expression in the motor cortex of the adult rat after motor skill learning. J Neurosci 16:4529–4535. 8699262 10.1523/JNEUROSCI.16-14-04529.1996 PMC6578852

[B44] Kleim JA, Barbay S, Nudo RJ (1998) Functional reorganization of the rat motor cortex following motor skill learning. J Neurophysiol 80:3321–3325. 9862925 10.1152/jn.1998.80.6.3321

[B45] Lefebvre V, Li P, De Crombrugghe B (1998) A new long form of Sox5 (L-Sox5), Sox6 and Sox9 are coexpressed in chondrogenesis and cooperatively activate the type II collagen gene. EMBO J 17:5718–5733. 10.1093/emboj/17.19.5718 9755172 PMC1170900

[B46] Lesnikova A, Casarotto PC, Fred SM, Voipio M, Winkel F, Steinzeig A, Antila H, Umemori J, Biojone C, Castrén E (2021) Chondroitinase and antidepressants promote plasticity by releasing TRKB from dephosphorylating control of PTPσ in parvalbumin neurons. J Neurosci 41:972–980. 33293360 10.1523/JNEUROSCI.2228-20.2020 PMC7880295

[B47] Lin Y, Bloodgood BL, Hauser JL, Lapan AD, Koon AC, Kim TK, Hu LS, Malik AN, Greenberg ME (2008) Activity-dependent regulation of inhibitory synapse development by Npas4. Nature 455:1198–1204. 18815592 10.1038/nature07319 PMC2637532

[B48] Lin PY, Kavalali ET, Monteggia LM (2018) Genetic dissection of presynaptic and postsynaptic BDNF-TrkB signaling in synaptic efficacy of CA3-CA1 synapses. Cell Rep 24:1550–1561. 30089265 10.1016/j.celrep.2018.07.020 PMC7176480

[B49] Lourenço J, Pacioni S, Rebola N, Van Woerden GM, Marinelli S, Digregorio D, Bacci A (2014) Non-associative potentiation of perisomatic inhibition alters the temporal coding of neocortical layer 5 pyramidal neurons. PLoS Biol 12:e1001903. 25003184 10.1371/journal.pbio.1001903 PMC4086817

[B50] Mardinly AR, Spiegel I, Patrizi A, Centofante E, Bazinet JE, Tzeng CP, Mandel-Brehm C, Harmin DA, Adesnik H, Fagiolini M, Greenberg ME (2016) Sensory experience regulates cortical inhibition by inducing IGF1 in VIP neurons. Nature 531:371–375. 26958833 10.1038/nature17187 PMC4823817

[B51] Micheva KD, Beaulieu C (1996) Quantitative aspects of synaptogenesis in the rat barrel field cortex with special reference to GABA circuitry. J Comp Neurol 373:340–354. 10.1002/(SICI)1096-9861(19960923)373:3<340::AID-CNE3>3.0.CO;2-2 8889932

[B52] Miyoshi G, Hjerling-Leffler J, Karayannis T, Sousa VH, Butt SJ, Battiste J, Johnson JE, Machold RP, Fishell G (2010) Genetic fate mapping reveals that the caudal ganglionic eminence produces a large and diverse population of superficial cortical interneurons. J Neurosci 30:1582–1594. 10.1523/JNEUROSCI.4515-09.2010 20130169 PMC2826846

[B53] Moore AK, Weible AP, Balmer TS, Trussell LO, Wehr M (2018) Rapid rebalancing of excitation and inhibition by cortical circuitry. Neuron 97:1341–1355.e6. 29503186 10.1016/j.neuron.2018.01.045 PMC5875716

[B54] Murakami A, Ishida S, Thurlow J, Revest JM, Dickson C (2001) SOX6 binds CtBP2 to repress transcription from the Fgf-3 promoter. Nucleic Acids Res 29:3347–3355. 11504872 10.1093/nar/29.16.3347 PMC55854

[B55] Nowicka D, Soulsby S, Skangiel-Kramska J, Glazewski S (2009) Parvalbumin-containing neurons, perineuronal nets and experience-dependent plasticity in murine barrel cortex. Eur J Neurosci 30:2053–2063. 20128844 10.1111/j.1460-9568.2009.06996.x

[B56] Ohba S, Ikeda T, Ikegaya Y, Nishiyama N, Matsuki N, Yamada MK (2005) BDNF locally potentiates GABAergic presynaptic machineries: target-selective circuit inhibition. Cereb Cortex 15:291–298. 15238431 10.1093/cercor/bhh130

[B57] Okaty BW, Miller MN, Sugino K, Hempel CM, Nelson SB (2009) Transcriptional and electrophysiological maturation of neocortical fast-spiking GABAergic interneurons. J Neurosci 29:7040–7052. 19474331 10.1523/JNEUROSCI.0105-09.2009 PMC2749660

[B58] Pangratz-Fuehrer S, Hestrin S (2011) Synaptogenesis of electrical and GABAergic synapses of fast-spiking inhibitory neurons in the neocortex. J Neurosci 31:10767–10775. 10.1523/JNEUROSCI.6655-10.2011 21795529 PMC3159030

[B59] Porcher C, Medina I, Gaiarsa JL (2018) Mechanism of BDNF modulation in GABAergic synaptic transmission in healthy and disease brains. Front Cell Neurosci 12:273. 30210299 10.3389/fncel.2018.00273 PMC6121065

[B60] Pulimood NS, Rodrigues WDSJ, Atkinson DA, Mooney SM, Medina AE (2017) The role of CREB, SRF, and MEF2 in activity-dependent neuronal plasticity in the visual cortex. J Neurosci 37:6628–6637. 28607167 10.1523/JNEUROSCI.0766-17.2017 PMC5508254

[B61] Rico B, Xu B, Reichardt LF (2002) TrkB receptor signaling is required for establishment of GABAergic synapses in the cerebellum. Nat Neurosci 5:225–233. 11836532 10.1038/nn808 PMC2758226

[B62] Rodriguez-Tornos FM, San Aniceto I, Cubelos B, Nieto M (2013) Enrichment of conserved synaptic activity-responsive element in neuronal genes predicts a coordinated response of MEF2, CREB and SRF. PLoS One 8:e53848. 23382855 10.1371/journal.pone.0053848 PMC3561385

[B63] Sánchez-Huertas C, Rico B (2011) CREB-dependent regulation of GAD65 transcription by BDNF/TrkB in cortical interneurons. Cereb Cortex 21:777–788. 20739478 10.1093/cercor/bhq150

[B64] Spiegel I, Mardinly AR, Gabel HW, Bazinet JE, Couch CH, Tzeng CP, Harmin DA, Greenberg ME (2014) Npas4 regulates excitatory-inhibitory balance within neural circuits through cell-type-specific gene programs. Cell 157:1216–1229. 24855953 10.1016/j.cell.2014.03.058 PMC4089405

[B65] Stolt CC, Schlierf A, Lommes P, Hillgartner S, Werner T, Kosian T, Sock E, Kessaris N, Richardson WD, Lefebvre V, Wegner M (2006) SoxD proteins influence multiple stages of oligodendrocyte development and modulate SoxE protein function. Dev Cell 11:697–709. 17084361 10.1016/j.devcel.2006.08.011

[B66] Swanwick CC, Murthy NR, Kapur J (2006) Activity-dependent scaling of GABAergic synapse strength is regulated by brain-derived neurotrophic factor. Mol Cell Neurosci 31:481–492. 16330218 10.1016/j.mcn.2005.11.002 PMC2842119

[B67] Takesian AE, Hensch TK (2013) Balancing plasticity/stability across brain development. Prog Brain Res 207:3–34. 24309249 10.1016/B978-0-444-63327-9.00001-1

[B68] Tan S, Xiao Y, Yin HH, Chen AI, Soong TW, Je HS (2018) Postnatal TrkB ablation in corticolimbic interneurons induces social dominance in male mice. Proc Natl Acad Sci USA 115:E9909–E9915. 30282736 10.1073/pnas.1812083115 PMC6196485

[B69] Tyssowski KM, Destefino NR, Cho JH, Dunn CJ, Poston RG, Carty CE, Jones RD, Chang SM, Romeo P, Wurzelmann MK, Ward JM, Andermann ML, Saha RN, Dudek SM, Gray JM (2018) Different neuronal activity patterns induce different gene expression programs. Neuron 98:530–546.e11. 29681534 10.1016/j.neuron.2018.04.001 PMC5934296

[B70] Van Welie I, Van Hooft JA, Wadman WJ (2004) Homeostatic scaling of neuronal excitability by synaptic modulation of somatic hyperpolarization-activated Ih channels. Proc Natl Acad Sci USA 101:5123–5128. 15051886 10.1073/pnas.0307711101 PMC387384

[B71] Wonders CP, Taylor L, Welagen J, Mbata IC, Xiang JZ, Anderson SA (2008) A spatial bias for the origins of interneuron subgroups within the medial ganglionic eminence. Dev Biol 314:127–136. 18155689 10.1016/j.ydbio.2007.11.018 PMC2727678

[B72] Wu X, Fu Y, Knott G, Lu J, Di Cristo G, Huang ZJ (2012) GABA signaling promotes synapse elimination and axon pruning in developing cortical inhibitory interneurons. J Neurosci 32:331–343. 22219294 10.1523/JNEUROSCI.3189-11.2012 PMC3742883

[B73] Xenos D, Kamceva M, Tomasi S, Cardin JA, Schwartz ML, Vaccarino FM (2018) Loss of TrkB signaling in parvalbumin-expressing basket cells results in network activity disruption and abnormal behavior. Cereb Cortex 28:3399–3413. 28968898 10.1093/cercor/bhx173 PMC6132287

[B74] Xue M, Atallah BV, Scanziani M (2014) Equalizing excitation-inhibition ratios across visual cortical neurons. Nature 511:596–600. 25043046 10.1038/nature13321 PMC4117808

[B75] Zheng K, An JJ, Yang F, Xu W, Xu ZQD, Wu J, Hökfelt TGM, Fisahn A, Xu B, Lu B (2011) TrkB signaling in parvalbumin-positive interneurons is critical for gamma-band network synchronization in hippocampus. Proc Natl Acad Sci USA 108:17201–17206. 21949401 10.1073/pnas.1114241108 PMC3193255

